# Sunbed Use among 11- to 17-Year-Olds and Estimated Number of Commercial Sunbeds in England with Implications for a ‘Buy-Back’ Scheme

**DOI:** 10.3390/children8050393

**Published:** 2021-05-14

**Authors:** Louisa G. Gordon, Rob Hainsworth, Martin Eden, Tracy Epton, Paul Lorigan, Megan Grant, Adéle C. Green, Katherine Payne

**Affiliations:** 1 Population Health Department, QIMR Berghofer Medical Research Institute, Brisbane Q4006, Australia; Adele.Green@qimrberghofer.edu.au; 2 School of Nursing, Queensland University of Technology (QUT), Brisbane Q4059, Australia; 3 School of Public Health, University of Queensland, Brisbane Q4006, Australia; 4 Manchester Centre for Health Economics, University of Manchester, Manchester M13 9PL, UK; rob.hainsworth@manchester.ac.uk (R.H.); Martin.Eden@manchester.ac.uk (M.E.); Megan.Grant@cruk.manchester.ac.uk (M.G.); katherine.payne@manchester.ac.uk (K.P.); 5 Manchester Centre for Health Psychology, University of Manchester, Manchester M13 9PL, UK; Tracy.Epton@manchester.ac.uk; 6 Cancer Research UK Manchester Institute, University of Manchester & Christie NHS Foundation Trust, Manchester M13 9PL, UK; paul.lorigan@nhs.net; 7 Division of Cancer Sciences, University of Manchester & Christie NHS Foundation Trust, Manchester M13 9PL, UK

**Keywords:** indoor tanning, sunbeds, children, prevalence

## Abstract

Prior to 2011 legislation prohibiting children from using commercial sunbeds, the prevalence of sunbed use in 15- to 17-year-olds in some areas in England was as high as 50%. Despite significant decreases since 2011, children today still practice indoor tanning. We estimated current sunbed use in 11- to 17-year-olds in England, the number of available commercial sunbed units, and the associated cost of a ‘buy-back’ scheme to remove commercial sunbeds under a potential future policy to ban sunbeds. We undertook a calibration approach based on published prevalence rates in English adults and other sources. Internet searches were undertaken to estimate the number of sunbed providers in Greater Manchester, then we extrapolated this to England. Estimated mean prevalence of sunbed use was 0.6% for 11- to 14-year-olds and 2.5% for 15- to 17-year-olds, equating to 62,130 children using sunbeds in England. A predicted 2958 premises and 17,865 sunbeds exist nationally and a ‘buy-back’ scheme would cost approximately GBP 21.7 million. Public health concerns remain greatest for 11- to 17-year-olds who are particularly vulnerable to developing skin cancers after high ultraviolet exposure.

## 1. Introduction

Emitted ultraviolet radiation (UVR) from indoor tanning devices such as sunbeds or tanning lamps can reach high to extreme levels [1]. To protect children from harmful exposure to UVR and prevent keratinocyte skin cancers and melanomas, England prohibited commercial sunbed use among under 18-year-olds in 2011. Commercial sunbeds relate to stand-alone premises and those located within gymnasiums and leisure centres or health clubs. Prior to 2011, the average prevalence of sunbed use across England was reported to be 6% in 11- to 17-year-olds but as high as 50% in 15- to 17-year-old girls in some northern cities [2]. Furthermore, over 50% of children using sunbeds reported they had suffered burns and 25% reported unsupervised use [2]. 

Since the 2009 classification of indoor tanning devices as carcinogenic to humans by the International Agency for Research on Cancer (IARC) [3], over 20 countries have now legislated against indoor tanning in persons aged under 18 years (or lower) [4]. Going further, Australia, Brazil and Iran have outright bans on all commercial indoor tanning services [5]. Other countries have introduced various restrictions such as preventing indoor tanning by UV-sensitive people, banning unsupervised access, licensing indoor tanning establishments, mandating operator training and taxing indoor tanning sessions. 

The combined influence of the IARC statement [3], consumer educational campaigns and warning notices [6] has led to sunbed use among children aged 11 to 17 years across Europe to nearly halve from 12.5% [4]. Despite this favourable trend, prevalence at 6.7% (95% Confidence Interval (CI): 4.4 to 9.6%) [4] remains concerning and banning use by children may not go far enough in deterring sunbed use in those < 18 years. In the United States, there are high levels of indoor tanning among 18-year-olds and college students when indoor tanning becomes legal [7]. The current extent of sunbed use among children in England is unknown but is likely to continue while the commercial provision of indoor tanning remains available to adults. Adolescents will side-step legislation and use sunbeds if regulatory enforcement of the law is lax [8], or when their parents allow them to use privately-owned sunbeds in their homes [9]. In a school environment, older peers and social media may also exert influence on their younger classmates [7], as well as the effects of wider media and social media.

Over 15,000 people in the United Kingdom are currently diagnosed with melanoma each year and there have been over 2000 deaths annually since 2007 [10]. On average, the number of new cases of melanoma in the UK among young people aged up to 24 years is 170 and 161 for keratinocyte skin cancer, rising to 482 and 505, respectively, in those aged up to 29 years [10]. The incidence of melanoma has increased 30% since 2007 [10] and calls to ban all commercial tanning have reignited [7]. Such planned legislation would likely meet with resistance from commercial operators and other interested parties who claim consumer benefits of sunbed use, claims that have been dismissed by the World Health Organization’s (WHO) report: Artificial tanning devices: public health interventions to manage sunbeds [6]. 

Primary motivating factors as to why young people engage in tan-seeking behaviours relate to aesthetics, the desire to look tanned and to feel better and more confident with perceived attractiveness associated with tanned skin (Eden et al. forthcoming). Females between the ages of 12 to 30 years are the key users of sunbeds [2,4] and commercial tanning outlets offer cheap and easy access to tanning facilities. These perceptions, coupled with an impetus for use ahead of milestone birthdays, end-of-school formal events or getting together with friends while tanning [11], create a socially acceptable demand for indoor tanning. Quantifying the current scale of sunbed provision identifies a baseline against which to assess the impact of any future ban on sunbed providers and informs the potential cost of a ‘buy-back’ scheme. A ‘buy-back’ approach has been successful in Australia [12] in transitioning to a legislative ban, encouraging the removal of sunbeds from the market and financially compensating sunbed providers for enforced changes to their business. In light of this, the purpose of this study was to estimate: the prevalence of sunbed use among children in England, the number of commercial tanning units and the costs of a potential ‘buy-back’ scheme.

## 2. Materials and Methods

No ethical approval was required as this study did not involve data collection but used existing published data.

Sunbed use prevalence: Gender-specific sunbed use prevalence at each age between 11 and 17 years was estimated based on numerous known and informed variables for England in 2020 [2,13,14,15]. For the purposes of derivation: prevalence for an age *x* was defined as the proportion of people having ever used a sunbed by their *x*th birthday; incidence for an age *x* was defined as the proportion of people using sunbeds for the first time when they are *x* years old. Sunbed use prevalence amongst 11–14-, 15–17- and 11–17-year-olds was calculated based on mid-year estimates for the relevant years.

Incidence was assumed to rise between the ages of 11 and 17 years. Following precedents in the literature [14], each year’s incidence was modelled as a multiple of the previous year’s. To ensure the 11- to 17-year-old model was consistent with sunbed prevalence estimates for young adults [15], a surge in incidence was also modelled at the 18-year threshold when sunbeds become legal in England using a one-off multiplier and the resulting young adult prevalence compared with published estimates. A published ratio of female-to-male use was used to apportion overall use between adolescent women and men [15].

Estimates of sunbed use prevalence reported from a recent meta-analysis (24) and from a Europe-wide study [15] reveal a declining trend in use since IARC’s 2009 UVR-carcinogen statement [3]. Comparing estimates from the two studies suggests European sunbed use is falling by 14% every two years. Published point estimates for 15- to 19-year-old use in England from before the 2010 ban on sunbeds for under 18-year-olds [2] were projected to 2020 based on this two-yearly proportional decrease. Comparing the English projected estimate with <20-year-old European prevalence for 2020, extrapolated using the same trend from the 2012 and 2014 estimates, indicated that the ban further reduced sunbed use in adolescents by 39 percentage points [14,16].

Estimates of sunbed use prevalence amongst 11–14, 15–17 and 11–17-year-olds from 2008 were projected to 2020 based on the downward European trend and effect of the English ban [2]. The confidence intervals of the adjusted estimates were used as targets to calibrate the parameters of a sunbed use prevalence curve for English adolescents. Sunbed use was assumed to be at any indoor tanning location, either in a commercial facility or at home. The following parameters and plausible sensitivity analysis values (in brackets) were used to plot the final curve:

- 11-year-old prevalence of 0.1% (0.0, 0.2%) and incidence of 0.2% (0.1, 0.3%);
- Multiplicative year-on-year increase in incidence between ages 11–17 of 33.3% (23, 43%);
- Female:male ratio of 2.11:1, from a published Irish sample of both adults and adolescents [15].

Estimated number of commercial sunbed units in England: We assessed two populations, Greater Manchester and the whole of England. Sunbed unit prevalence in Greater Manchester was chosen as the locality to inform extrapolation to the whole of England. Greater Manchester is a large metropolitan county, about one-third the population size of Greater London, and comprising a mix of rural and urban residential areas. It contains ten local authorities; two cities (Manchester and Salford) along with eight metropolitan boroughs. Greater Manchester is socio-economically diverse with areas of both low and high deprivation. Therefore, sensitivity analyses were undertaken for low and high values for the estimated number of beds per provider.

Google and Google Maps searches for sunbed providers in each of the 10 local authorities of Greater Manchester were undertaken in April 2020 [17,18]. Information from one national chain of sunbed shops and two national health club chains was also used. Commercial sunbed providers were classified as either small sunbed shop or large sunbed shops. For each of these types of providers, an assumption was made about the typical number of beds provided per commercial provider based on information from sunbed users who were close contacts of the research team. To extrapolate a figure for England, a per-person sunbed annual rate was subsequently calculated by dividing the estimated number of sunbeds in Greater Manchester by the population of all people living in the area in 2019 (*n* = 2,835,686) [19]. This annual rate was applied to the population of England in 2019 (*n* = 56,287,000) [19]. Sensitivity values for the number of sunbeds per different type of premise were used to calculate a range of plausible estimates.

### Cost of a ‘Buy-Back’ Scheme 

In a potential ‘buy-back’ scheme, all current commercial providers of sunbeds would be offered a per-sunbed amount of compensation ahead of the ban to have sunbeds removed from their premises. Three per-sunbed unit costs were calculated based on the different amounts used in three existing Australian state-based buy-back schemes in 2014 (AUD 1000, 2000 or 5000 (C. Sinclair, personal communication)). These costs were inflated to 2020 prices [20] and translated into GBP using the https://www.xe.com/currency converter (August 2020). The respective three exemplar per-unit buy-back costs were then: GBP 603, GBP 1207 or GBP 3017. 

## 3. Results

### 3.1. Sunbed Use Prevalence

**Figure 1:**
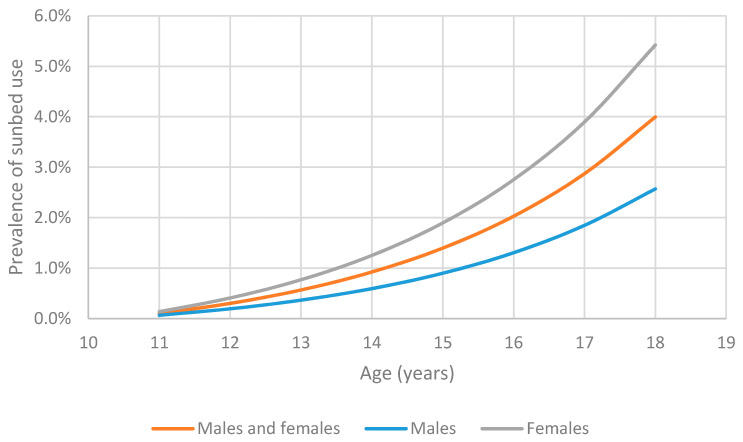
Percentage of 11- to 17-year-olds estimated to use sunbeds in England.

The estimated mean prevalence of sunbed use overall was 1.5% for 11 to 17 years, comprising 0.6% in 11- to 14-year-olds and 2.5% in 15- to 17-year-olds (Figure 1). Prevalence of sunbed use for 11–14-year-old females was 0.9%, and 0.4% for males, compared with 15–17-year-old females at 3.4% and males at 1.6%. Applying this to the population of children in England aged 11 to 17 years, by age and gender, equates to 62,130 children estimated to be using sunbeds. Sensitivity analyses show that the overall prevalence may vary between 0.8 to 2.1% (Table 1) and this is mostly driven by the change in incidence rate per year of age.

### 3.2. Estimated Number of Commercial Sunbed Units in England

The number of beds per provider were six beds for a small shop and eight for a larger shop. In Greater Manchester, there were an estimated 900 commercial sunbeds in current use (Table 2). Extrapolating from a per-capita Greater Manchester figure (1 sunbed per 3152 residents), there are an estimated 17,865 commercial sunbed devices across England. With an average of 6.04 sunbeds per commercial provider based on Greater Manchester assumptions, we estimated that England has 2958 commercial sunbed providers (17,865 sunbed units) in operation. Sensitivity analyses showed the number of providers for England could vary between 1518 and 3917, translating to a total number of sunbed devices between 9170 and 23,661.

### 3.3. Cost of a ‘Buy-Back’ Scheme 

Three estimated ‘buy-back’ scheme costs from both a regional and national perspective are detailed in Table 3. For Greater Manchester, depending on unit costs used, a scheme could cost between nearly GBP 550k up to almost GBP 2.8 million for the total sunbed units. From a national perspective, it is predicted that a scheme would cost GBP 21.7 million, or between ~GBP 11 million and ~GBP 55 million.

## 4. Discussion

Our study showed that the current prevalence of sunbed use among children in England may be around 1.5% (or over 62,000 individuals) and around 18,000 commercial sunbed units are likely to be in operation, awaiting uptake of indoor tanning when these children turn 18 years old. Girls are twice as likely to use sunbeds as boys, but for both sexes, sunbed use substantially rises in the 15–17-year-old age groups. Since exposure to UVR is the predominant cause of keratinocyte skin cancers and melanoma [3], these cancers are largely preventable by reducing personal exposure to UV radiation through sun protection [21] or avoiding indoor tanning [22]. Early onset melanoma in youth is serious and the most frequently diagnosed cancer in young adults [23]. With rising melanoma mortality rates every year in many countries, including England, more needs to be done to prevent this disease. Public health concerns are greatest for youths who are particularly vulnerable to skin cancers after high UV exposure [6]. The data presented here may be used in subsequent economic analyses of the impact of banning sunbeds and provide further evidence for the case for a ban on commercial sunbed services in England.

For perceived aesthetic reasons, indoor tanning is likely to continue to be a popular activity among youth in Europe, the Americas and Canada. While banning children’s sunbed use is a positive step to reduce the burden of skin cancers, these gains may be quickly reversed if parents are users of sunbeds and influence uptake in their teenage children. Furthermore, using a sunbed may be seen as safe once children are older if there is no legislation in place. In a large study in France, parents with a lower understanding of sun safety measures and less stringent protection behaviours had children engaging in fewer sun protection practices than children of parents who did practise sun protection [24]. Legislation banning commercial sunbeds sends a clear message from the government that they are protecting citizens’ health, and moreover it is highly likely to be an investment that will save healthcare costs in the longer-term [14,16].

A ‘buy-back’ scheme may cost the government upwards of GBP 55 million but notably, is a one-off investment for a permanent solution to remove access to young people, the primary users of sunbeds. It is feasible that resources are higher for auditing and enforcement of an under 18s ban compared with a complete ban, due to their ongoing nature and the substantial volume of sunbed businesses to manage. Compliance with age restriction legislation prior to 2011 was poor in Chile, the US and Australia, with compliance at just 34% (mean) after in-person inquiries (range 20–62%) [8]. Even if compliance to the current under 18s ban in England were 80%, and assuming our estimated 1.5% prevalence to be home-use only, a total of 77,663 (62,130/0.80) 11- to 17-year-olds could be using sunbeds across England. While non-compliance to a full ban is also possible, it was demonstrated to be a minor and temporary problem in the state of Victoria, Australia [12]. A further benefit of banning sunbeds is the avoidance of serious blistering and erythema, and potential hospitalizations that occur for sunbed-induced burns [13,25], as well as avoidance of eye damage [6]. 

Estimating the number of commercial sunbed units in Greater Manchester was challenging. A geographical gradient in England is evident in sunbed use with northern England having higher sunbed use than southern areas [2]. The estimated number of providers in Greater Manchester is likely to be a conservative estimate because not all providers will have been identified using Google and Google Maps searches. Stark disparities in the identified number of providers in the neighbouring (and similarly sized in terms of population) local authorities provides an indication of the potential underestimation. Whilst Greater Manchester is socioeconomically and geographically diverse, we acknowledge that caution should be exercised in interpreting the extrapolated England-wide estimate, given previously reported regional variation in sunbed use [2]. Further research could be conducted within a sample of English local authorities to triangulate these findings.

In calculating the ‘buy-back’ scheme cost estimates, several assumptions have necessarily been made as mentioned above regarding the estimated numbers of sunbed providers. The Sunbed Association’s website states the organization represents “a significant section of the tanning facilities in the UK and Ireland” (https://www.sunbedassociation.org.uk/), but membership of the Association is voluntary so does not provide a definitive list of all UK sunbed providers. In a decade-old report, the South West Public Health Observatory’s Cancer Intelligence Service estimated the number of facilities for sunbed use in the UK was a total of 5350. Within that report, the Sunbed Association provided a personal communication with their estimation that approximately 8000 sunbed providers were in operation during 2006. By attaching a plausible range of unit costs to the number of commercial sunbeds, an early indicative overview of the potential impact of buy-back schemes in the England context has been provided. In Australia, ‘buy-back’ schemes were undertaken to remove commercial sunbeds from premises during the time that bans were enforced. For example, in Victoria, Australia, ahead of the 2014 ban, operators were offered AUD 2000 (approximately GBP 1200 today) for each unit. Funded by the Australian government, it was estimated to cost between AUD 400,000 and AUD 750,000 (GBP 250 k to GBP 457 k) for the scheme in the state of Victoria alone [26]. However, these upfront costs are equivalent in value to the avoided incidence of only five or six patients with advanced stage melanoma and the attendant very high costs of immunotherapy treatments.

Factors other than geographic location, gender and legislation influence sunbed use among teenagers and they need to be further explored by future studies. Low self-confidence, high social pressure and cultural beauty standards prompt individuals to use a sunbed for the first time [27]. The identification of the most susceptible subgroups to indoor tanning among the general population is key to ensure successful outcomes from campaigns targeting sunbed use. Other successful contributors to introducing sunbed legislation include measures to counteract the strong marketing strategies from the tanning bed industry and anti-‘nanny state’ attitudes, while enhancing the need for strong advocacy and key influencers (e.g., dermatologists in favour of banning sunbeds) and addressing other methods of increasing confidence among children.

## 5. Conclusions

Sunbed use among children aged 11- to 14-years-old in England is estimated to be 0.6% per year, rising to 2.5% among 15- to 17-year-olds. This translates to potentially 62,000 children across England engaging in indoor tanning, known to be harmful and cause skin cancers in users. The estimated number of commercial sunbed devices is approximately 18,000 in England. These findings provide the basis for subsequent economic analyses of the impact of banning commercial sunbeds in England.

## Figures and Tables

**Table 1:** One-way sensitivity analyses of prevalence of sunbed use in 11- to 17-year-olds.

	Mean by Age Group		Overall
	11–14-Year-Olds	15–17-Year-Olds	11–17-Year-Olds
Base case	0.63%	2.53%	1.45%
11-year-old prevalence (base 0.10%)			
0.00%	0.53%	2.43%	1.35%
0.20%	0.73%	2.63%	1.55%
11-year-old incidence (base 0.2%)			
0.10%	0.37%	1.31%	0.77%
0.30%	0.90%	3.74%	2.12%
Increase in incidence per year (base 33.3%)			
23%	0.59%	2.00%	1.19%
43%	0.68%	3.16%	1.74%

**Table 2:** Estimated number of providers of commercial sunbeds and units in Greater Manchester.

Type of Sunbed Provider	Number of Providers ^1^	Units per Provider (Sensitivity Values)	Estimated no. of Sunbeds	Sensitivity Results Estimated no. of Sunbeds
Small sunbed shops	146	6 (3, 8)	876	438,1168
Large sunbed shops	3	8 (7, 12)	24	21,36
Total		Mean 6.04 (3.10, 6.12)	900	462,1192

^1^. Using data for April 2020. Source: small sunbed shops have six devices, larger sunbed shops are part of chains.

**Table 3:** Estimated cost of buy-back schemes.

Location	Unit Cost of Sunbed (GBP 2020)	No. of Sunbeds	Unit Cost of Removal	No. of Premises	Total Cost of Buy-Back Scheme
Greater Manchester	603	900	GBP 40 *	149	GBP 548,660
	1207				GBP 1,092,260
	3017				GBP 2,721,260
England	603	17,865	GBP 40 *	2958	GBP 10,890,915
	1207				GBP 21,681,375
	3017				GBP 54,017,025

* Assumption based on local council charges for removing large appliances (https://www.salford.gov.uk).

## Data Availability

Data supporting the reported results can be accessed by reasonable request to the authors.
